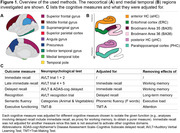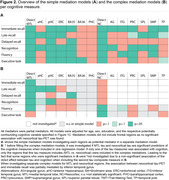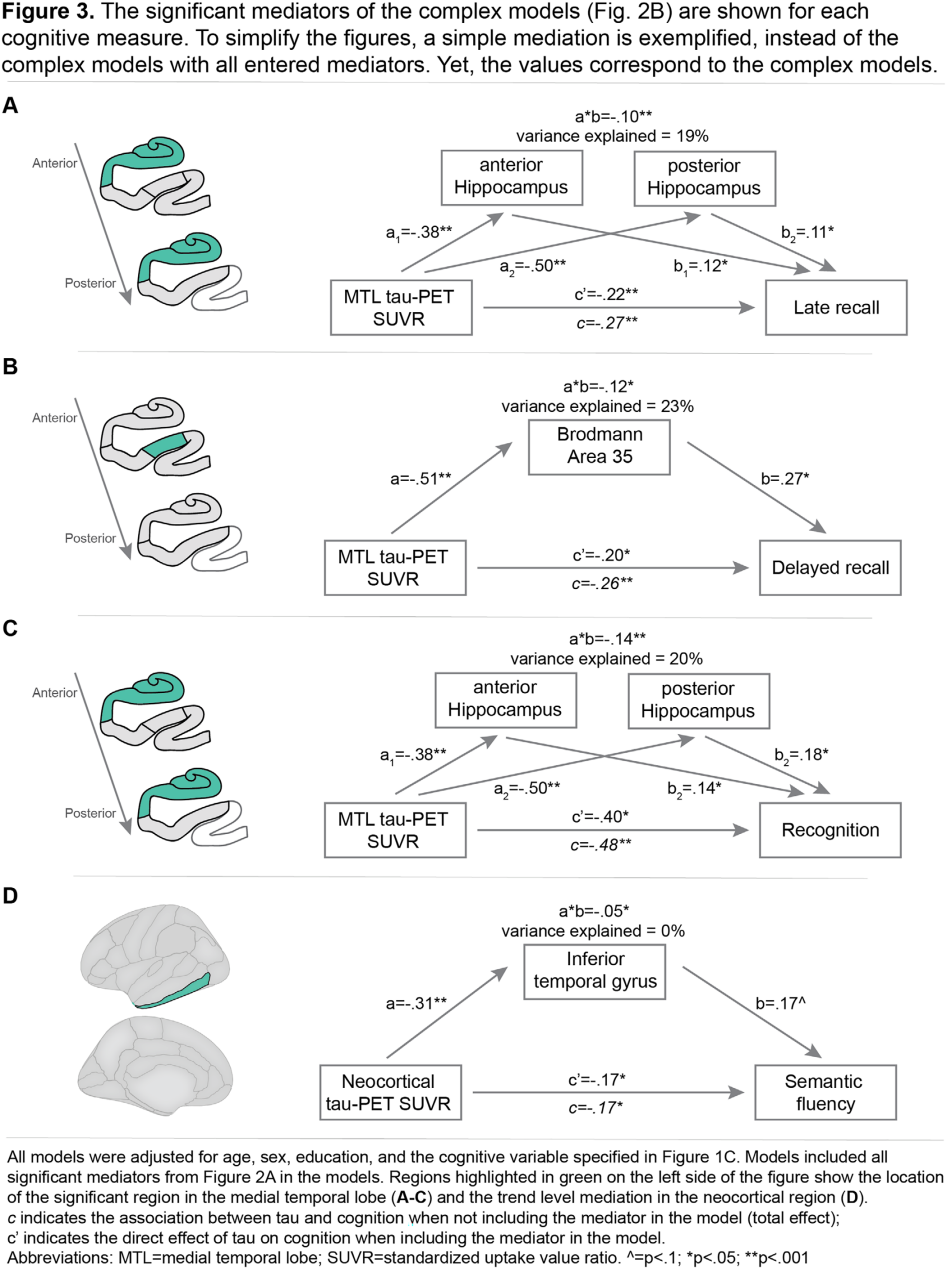# The association between tau pathology and later stages of memory processing is mediated by medial temporal lobe atrophy in typical Alzheimer’s Disease

**DOI:** 10.1002/alz.087337

**Published:** 2025-01-03

**Authors:** Anika Wuestefeld, Salena Cui, Sandhitsu R. Das, Long Xie, Emily McGrew, Paul A. Yushkevich, Laura Wisse, David A Wolk, the Alzheimer’s Disease Neuroimaging Initiative

**Affiliations:** ^1^ Lund University, Lund Sweden; ^2^ University of Pennsylvania, Philadelphia, PA USA; ^3^ Siemens Healthineers, Princeton, NJ USA; ^4^ Perelman School of Medicine, University of Pennsylvania, Philadelphia, PA USA

## Abstract

**Background:**

Tau pathology is closely related to cognitive decline in Alzheimer’s disease (AD). Neurodegeneration is the putative mechanism by which tau leads to cognitive deficits. However, there is limited work on the associations between tau, neurodegeneration, and specific cognitive domains. We investigate potential mediating effects of medial temporal lobe (MTL) and neocortical neurodegeneration for different cognitive domains in amyloid‐β‐positive (Aβ+) adults.

**Method:**

We included 325 positron emission tomography (PET) Aβ+ individuals across the AD spectrum from the Alzheimer’s Disease Neuroimaging Initiative (age: 72±8 [55–90]; 51.1% female, education: 16±2 years). Hippocampal volume and cortical thickness were measured from T1‐weighted structural magnetic resonance imaging in typical AD regions (Fig. 1). Using flortaucipir‐PET, tau was quantified for two composite regions (MTL, neocortical). Included cognitive measures are shown in Fig. 1. Mediation analyses between tau‐PET, thickness/volume, and cognition were performed. Cognitive measures were adjusted to isolate the function of interest (Fig. 1).

**Result:**

Higher tau‐PET uptake and lower thickness were associated with lower performance on all cognitive measures.

In simple mediations, tau‐immediate recall associations were mediated by MTL, parietal, and temporal regions (Fig. 2A). Tau‐delayed recall associations were mediated by MTL regions. Tau‐late recall and tau‐recognition associations were mediated by MTL and parietal regions. Tau‐fluency and tau‐executive functioning associations were mediated by MTL and parietal regions, as well as temporal regions for fluency.

Complex mediations investigated significant mediators simultaneously (Fig. 2B+3). Depending if MTL/neocortical tau remained significant when covarying for the other tau measure in a regression, the complex mediation models focused on MTL and/or neocortical regions (Fig. 2B description). Hippocampus mediated the tau‐late recall and tau‐recognition associations. Brodmann area 35 mediated the tau‐delayed recall association. Tau‐fluency was, at a trend level, mediated by inferior temporal thickness. Tau‐executive functioning did not demonstrate a mediation effect, potentially due to limited frontal atrophy. All mediators were partial mediators explaining up to 23% of variance.

**Conclusion:**

The results suggest that MTL tau pathology, partially mediated by macrostructural changes, lead to impairments in specific memory domains in early AD. Impairments in fluency and executive functioning, however, seem more likely to follow from tau‐induced effects in neocortical regions.